# Short term serum pharmacokinetics of diammine silver fluoride after oral application

**DOI:** 10.1186/1472-6831-12-60

**Published:** 2012-12-31

**Authors:** Elsa Vasquez, Graciela Zegarra, Edgar Chirinos, Jorge L Castillo, Donald R Taves, Gene E Watson, Russell Dills, Lloyd L Mancl, Peter Milgrom

**Affiliations:** 1 Area of Pediatric Dentistry, Universidad Catolica Santa Maria de Arequipa, San Jose S/N, Arequipa, Peru; 2 Private practice in Arequipa, Garcilazo de la Vega 102- Umacollo, Arequipa, Peru; 3 Department of Dentistry for Children and Adolescents, Universidad Peruana Cayetano Heredia, Av. Honorio Delgado 430, Lima 31, Peru; 4 Department of Oral Health Sciences, Northwest Center to Reduce Oral Health Disparities, University of Washington, Box 357475, Seattle, WA, 98195-7475, USA; 5 Eastman Institute for Oral Health, University of Rochester, Rochester, NY, 14642, USA; 6 Department of Environmental Health, University of Washington, Seattle, WA, 98195-7234, USA

**Keywords:** Acute pain, Tooth, Medical device, Topical agent, Pharmacology, Toxicology

## Abstract

**Background:**

There is growing interest in the use of diammine silver fluoride (DSF) as a topical agent to treat dentin hypersensitivity and dental caries as gauged by increasing published research from many parts of the world. While DSF has been available in various formulations for many years, most of its pharmacokinetic aspects within the therapeutic concentration range have never been fully characterized.

**Methods:**

This preliminary study determined the applied doses (3 teeth treated), maximum serum concentrations, and time to maximum serum concentration for fluoride and silver in 6 adults over 4 h. Fluoride was determined using the indirect diffusion method with a fluoride selective electrode, and silver was determined using inductively coupled plasma-mass spectrometry. The mean amount of DSF solution applied to the 3 teeth was 7.57 mg (6.04 μL).

**Results:**

Over the 4 hour observation period, the mean maximum serum concentrations were 1.86 μmol/L for fluoride and 206 nmol/L for silver. These maximums were reached 3.0 h and 2.5 h for fluoride and silver, respectively.

**Conclusions:**

Fluoride exposure was below the U.S. Environmental Protection Agency (EPA) oral reference dose. Silver exposure exceeded the EPA oral reference dose for cumulative daily exposure over a lifetime, but for occasional use was well below concentrations associated with toxicity. This preliminary study suggests that serum concentrations of fluoride and silver after topical application of DSF should pose little toxicity risk when used in adults.

**Clinical trials registration:**

NCT01664871.

## Background

We previously demonstrated that diammine silver fluoride (DSF) applied topically to teeth of adults reduced the pain response to a cold air blast [1]. The pain reduction increased from 24 h to 7 days [1], and was much greater than with similar treatment with fluoride varnish [2]*.* Others have shown its effectiveness in root [3] and coronal dental caries [4]. It may also be a substitute for fissure sealants [5]. No adverse changes to teeth or intraoral tissues have been reported. Earlier, Gotjamanos and Ma [6] published an animal study suggesting that the high fluoride concentrations in one of these products could cause fluorosis.

Free silver ions are principally responsible for the antimicrobial action of diammine silver fluoride (DSF). It is widely known that silver ions denature enzymes of bacterial organisms by binding to reactive groups, resulting in their precipitation and inactivation [7,8]. Silver reacts with their thiol groups to form silver sulfides. Silver also reacts with the amino-, carboxyl-, phosphate-, and imidazole-groups and diminishes the activities of lactate dehydrogenase and glutathione peroxidase [9]. Bacteria are, in general, affected by this oligodynamic effect. Further, Knight and colleagues [10] have demonstrated that *Streptococcus mutans* is unable to form a biofilm on diammine silver fluoride-treated dentinal surfaces.

In early studies of inhibition and killing properties against oral bacteria, Thibodeau and colleagues [11] found silver ions were effective against broadly-infected dentin samples and several specific oral bacteria, including *S. mutans* GS-5, *R. dentocariosa* (ATCC 17931), *A. viscosus* (ATCC 15987), *V. alcalescens* (ATCC 17745), and *N. subflava*. Moreover, the minimum inhibitory concentrations of silver ions generated from multiple sources (i.e., from AgF, AgNO_3_, or electrically generated in solution) were equipotent [11] irrespective of the source, indicating that the antibacterial properties were solely dependent on the concentration of silver ions and not the specific compound or its other components. Tanzer and colleagues [12] reported that a single application of diammine silver fluoride topical treatment on rats with an established flora was associated with decrease of total recoverable facultative flora and *S. mutans* counts on teeth. At 62 days post-inoculation in this well-established animal model of human disease, the absolute recoveries of total flora and mutans streptococci were reduced by 34-47% (all p  <0.05) compared with groups receiving 5000 ppm F as sodium fluoride neutral gel or distilled water. These data are consistent with published *in vitro* studies [13,14].

Other than our own clinical study [1] and the animal study by Gotjamanos and Ma [6] no investigator has formally studied the safety of this widely used agent. The aim of this preliminary study was to characterize the short term pharmacokinetics of fluoride and silver in serum, subsequent to oral ingestion of DSF from topical application to teeth of adults.

## Methods

### Participants

Six healthy volunteers (4 females, mean age 36.2, range 23–52 y) were recruited from staff members of the Universidad Católica de Santa María, Arequipa, Peru. Each had gingival recession exposing non-carious cementum. Participants were taking no medications and had most permanent teeth present. Participants were asked to avoid fish and tea for 12 h before the study and not use fluoridated toothpaste in the preceding 4 hours. The Comité de Etica de Investigacion, Universidad Católica de Santa María approved the study and informed consent was obtained. The study registration is NCT01664871.

### Study agent

DSF [Ag(NH_3_)_2_ F, CAS RN 33040–28–7, Saforide, Toyo Seiyaku Kasei Co. Ltd. Osaka, Japan] was used. DSF is clear and colorless, with a weak odor of ammonia. The 38% solution contains between 24.4-28.8% (w/v) silver (Ag), and 5.0-5.9% fluoride (F). Concentrations of Ag and F in the DSF used for this study were 24.9% and 5.5%, respectively, and the specific gravity was 1.253 at 25°C (Certificate of Analysis Lot #155060 from the manufacturer). Diammine silver fluoride is also referred to in the literature as silver diammine fluoride, silver diamine fluoride or silver fluoride.

#### Procedures

Three maxillary teeth (cuspid and premolars) in the same quadrant were treated. Three teeth were treated because this was the average number of sensitive teeth reported in an epidemiological study of adults [15]. The teeth were free of restorations or cavitation. The facial (buccal) surface was dried with cotton gauze and then DSF was applied to the cervical area of each tooth. The teeth were isolated in order to avoid wetting the brush with saliva. Each tooth was coated using a separate microbrush, which was dipped in the DSF and weighed before and after the application using an analytical balance (Model S2000, Kern & Sohn GmbH, Balingen, Germany). The sum of the differences in weight of material applied was calculated for each participant. The teeth were not rinsed after application, however, the subjects were allowed to swallow as necessary during the 4 hour observation period.

### Blood collection

Immediately before application of DSF and then again at approximately 30 min, 1, 2, 3 and 4 h after application, blood was obtained from an antecubital vein. An attempt was made to collect at least 5 mL at each time point although the amount collected varied. The time periods were picked based on a previous pharmacokinetics study of fluoride varnish [16]. The blood was transferred to a fluoride-free plastic vacuum collection tube with clot activator (BD Vacutainer Plus Plastic Serum Tube, BD Diagnostics, NJ, USA). The tubes were spun at 1100 × g for 10 min and serum transferred by plastic pipette to 2 mL plastic cryogenic tubes. The samples were frozen and transported to the University of Washington for analysis.

### Analysis

#### Fluoride

Samples were thawed and analyzed in duplicate or triplicate using the diffusion and detection method with hexamethyldisiloxane (HMDS) and Orion F-sensitive electrode [17,18]. To improve recovery of the fluoride, the pre-diffusion step was omitted [19], and seals were completed before mixing 1 mL of 3 mol/L H_2_SO_4_-saturated HMDS with 1 mL of serum [20]. The trapping solution was 10 μL of 1 M NaOH. It was dried down to eliminate variability of evaporation and residual HMDS before adding 100 μL 3 mol/L acetic acid to a final pH 3.4. Standards were 10 and 100 μmol/L fluoride in the same final concentration of NaOH and acetic acid corresponding to 1 and 10 μmol/L F in the samples. ORION F and sleeve type reference electrodes were coupled with an Accumet Basic 0.1 mV meter (Thermo Fisher Scientific, Milwaukee WI, USA). Fluoride concentrations were averaged and are expressed as μmol/L.

Blood collection tubes were tested for fluoride by adding 3 mL of bovine plasma to 2 tubes and letting them set overnight before combining the 2 samples and comparing them with plasma directly placed in diffusion dishes. Analyses were carried out in triplicate and values averaged. Average concentrations (μmol/L ± SD) were 0.342 ± 0.014 and 0.320 ± 0.012, respectively (P > 0.1). The results show that less than 2% of the fluoride in the plasma samples came from the blood collection tubes and clot enhancer. In a separate study, recovery of fluoride (mean ± SD) of 1 nmol/mL F added to commercial bovine plasma, and analyzed using the same method, was 101 ± 4%.

#### Silver

Samples were thawed and analyzed one time using Inductively Coupled Plasma-Mass Spectrometry [U.S. Environmental Protection Agency (EPA) 6020a Rev.1 2007]. Serum (0.5 mL) was brought to 2 mL with dilute acid (final concentration: 2% HCl, 1% HNO3; trace metal grade, Fisher, Fairlawn, NJ, USA). Samples were centrifuged (1290 g) for 10 min and filtered (0.45 μm, PTFE, 13 mm [Whatman International Ltd, Kent, UK]; all polypropylene syringe) into 15 mL polypropylene centrifuge tubes. The samples were analyzed on an Agilent 7500CE (Santa Clara, CA, USA) inductively-coupled mass spectrometer (ICPMS) with He as the collision cell gas and an ASX-510 autosampler (CETAC, Omaha, NB, USA). A Micromist nebulizer (Glass Expansion, Pocasset, MA, USA) was used (1.15 L/min carrier gas; no makeup gas). Spray chamber temperature was 2°C. RF power was 1500 W. ^107^Ag was quantified using Y as internal standard. Calibrants (0, 0.5, 1, 5, 10, and 50 ng/mL) were in 2% HCl and 1% HNO3 and were prepared from commercial ICPMS grade solution (10,000 μg/mL, Ultra Scientific, N. Kingstown, RI, USA) by serial dilution. Concentration of calibrants was confirmed by a dilution of stock from a different manufacturer (BDH Aristar, VWR, Radnor, PA, USA). Calibration was by weighted (1/cps) linear regression. Instrument duplicates were run on 10% of samples; for instrument duplicates above reporting limit (N = 3) the average coefficient of variation was 2.8%. Data was corrected by the procedure blank. The level of detection was approximately 0.1 ng/mL. Values of silver less than 2 ng/mL were recorded to 2 (rounded to the same significance as the reporting limit). The amount of silver was converted to nmol (1 nmol = 107.8682 ng) and silver concentrations were expressed as nmol/L of serum.

### Soft tissue assessment

The gingiva adjacent to the application of DSF and the mucosa generally were observed at baseline and 24 h after treatment. Erythema, bleeding, white changes, ulceration and pigmentation were assessed using methods developed for an earlier study [1].

## Results

### Participants and amount of diammine silver fluoride applied

Table 1 gives the body weight of participants, weight/calculated volume of DSF applied to each participant’s 3 teeth, dose of F applied, and calculation of the current U.S. Environmental Protection Agency (EPA) oral reference dose (RfD) for daily F exposure based on the subject’s weight. The mean subject weight was 63, range 48–84 kg. Average total weight (calculated volume) of DSF applied was 7.57 mg (6.04 μL), corresponding to a mean application of 0.33 mg F based on the lot analysis.

**Table 1:** Body weight, amount of diammine silver fluoride (DSF) applied, fluoride dose, and serum fluoride pharmacokinetics in treatment of dentin hypersensitivity in adults

**Subject**	**Body weight (kg)**	**Weight of DSF solution applied (mg)**	**Calculated volume of DSF solution applied (μL)**^**a**^	**Calculated fluoride applied (mg)**^**b**^	**Fluoride - oral RfD**^**c**^**NOAEL**^**d**^**for subject (mg)**^**e**^	**Time to maximum serum concentration (h)**	**Maximum serum fluoride (μmol/L)**
1	48	3.8	3.03	0.17	2.88	3.0	1.46
2	54	8.2	6.54	0.36	3.24	3.2	2.88
3	84	5.8	4.63	0.25	5.04	4.0	1.02
4	82	7.6	6.07	0.33	4.92	3.0	2.77
5	54	11.9	9.50	0.52	3.24	1.0	1.56
6	56	8.1	6.46	0.36	3.36	4.0	1.45

^a^Calculated based on Specific Gravity of 1.253.

^b^Calculated based on volume of DSF solution applied times 0.055 mg/μL.

^c^RfD = U.S. Environmental Protection Agency oral reference dose.

^d^NOAEL = no observable adverse effect level.

^e^Calculated based on subject weight (kg) times NOAEL of 0.06 mg/(kg d).

Table 2 gives the dose of Ag applied and calculation of the current EPA oral reference dose (RfD) for daily Ag exposure for each subject, based on their weight. The mean total amount of Ag applied to the 3 teeth was 1.50 mg.

**Table 2:** Silver dose and serum silver pharmacokinetics after application of diammine silver fluoride topically

**Subject**	**Silver applied (mg)**^**a**^	**Silver - RfD**^**b**^**NOAEL**^**c**^**for subject (mg)**^**d**^	**Time to maximum serum concentration (h)**	**Maximum serum silver (nmol/L)**
1	0.75	0.24	2.0	28
2	1.63	0.27	2.1	269
3	1.15	0.42	4.0	250
4	1.51	0.41	3.0	213
5	2.37	0.27	1.0	269
6	1.61	0.28	3.0	204

^a^Calculated based on volume of DSF solution applied times 0.249 mg/μL.

^b^RfD = U.S. Environmental Protection Agency oral reference dose.

^c^ NOAEL = no observable adverse effect level.

^d^Calculated based on subject weight (kg) times NOAEL RfD of 0.005 mg/(kg d).

### Serum concentrations of fluoride and silver

**Figure 1:**
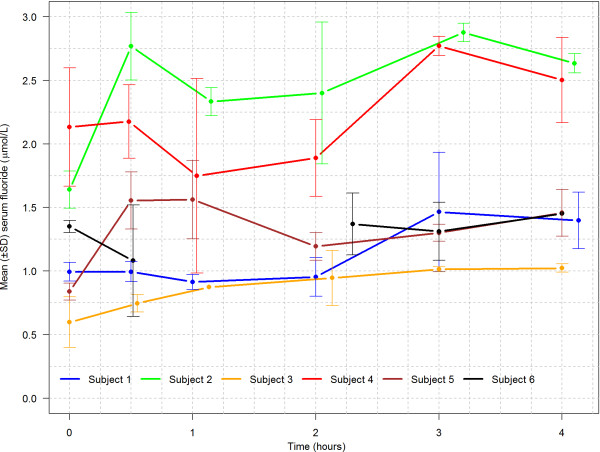
Mean (±SD) serum fluoride(μmol/L) after topical application of diammine silver fluoride to the facial (buccal) surfaces of 3 teeth in 6 adults.

**Figure 2:**
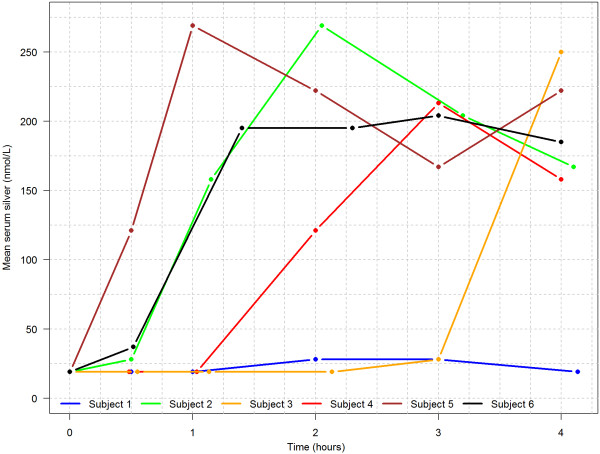
Silver values (nmol/L) after topical application of diammine silver fluoride to the facial (buccal) surfaces of 3 teeth in 6 adults.

Plots of the total concentrations of F and Ag in serum over time are shown in Figures 1 and 2. Serum at a single point was lost in one of the participants (Subject 6 at t = 1 h). The collection times vary among the participants as a result of practical considerations in collecting the samples. The mean maximum fluoride concentration attained during the 4 hour observation period was 1.86 μmol/L at 3.0 h after application of DSF. The mean maximum Ag concentration was 206 nmol/L reached after 2.5 h. Tables 1 and 2 give the individual maximum concentrations and time points for each participant.

### Safety

Subjects reported no discomfort from the topical treatment. No gingival or mucosal leukoplakia, inflammation, pigmentation or ulceration was observed in any subject immediately after treatment or at 24 h.

## Discussion

DSF is used topically to reduce dentin hypersensitivity in adults [1] and arrest tooth decay in both adults and children [4]. For sensitivity in adults, a single application reduces sensitivity by at least half [1] and application 2 or 3 times per year is sufficient clinically. In applying DSF, no deleterious changes in the gingiva were noted in our earlier study nor were changes seen in this work. Direct application of various silver compounds to wounds has been associated with localized argyria [21,22]. DSF should therefore be applied to teeth with care when used near areas of recent surgery, injury, or soft tissue erosion. DSF should also be used with caution in patients habitually consuming large amounts of colloidal or other compounds of silver [23]. DSF solutions will stain countertops and irreversibly damage clothing.

Maximum serum concentrations of fluoride did not exceed concentrations adults experience when using fluoridated toothpaste [16]. Fluoride exposure was below the EPA RfD [24]. Thus, the study results suggest that a single diammine silver fluoride treatment poses no toxic risk.

Oral exposure to metallic silver and numerous soluble silver compounds is common [25]. For instance, intraoral silver jewelry (piercings), and medical devices such as silver-coated intraurethral and intravascular catheters, and dental amalgam restorations can expose an individual to metallic silver. Silver and silver compounds (i.e. nitrates, chlorides, oxides, and sulfides of silver) can also be found in food and water. In its 1990 review of the toxicology of silver, ATSDR found no human studies of acute or chronic oral exposure to silver that resulted in death. Rat studies suggest the no observable adverse effect level (NOAEL) from ingested silver is greater than 181 mg/(kg day) for acute exposure of 14 continuous days (Table 2), [1,26]. This level is more than 75 times higher than the maximum amount of silver applied to the subject’s teeth in this study.

A recent case report [27] provides details of a patient who consumed a cumulative dose of 200 g silver and developed argyria, but was otherwise healthy. His consumption was approximately 648 mg of colloidal silver every day for 10 months. This is a daily dose of silver that is 275 times higher than the maximum amount applied as DSF in this study. DSF is intended for professional application no more than 2–3 times per year. When used as intended, the amount of silver applied as DSF should be well below the level to exhibit a toxic effect. While considered ‘cosmetic’ rather than toxic effect by the FDA [28], an irreversible blue or blue-gray discoloration of the skin or mucous membranes (argyria), or eyes (argyrosis) may develop with chronic exposure to silver and silver compounds. Evidence suggests silver salts (soluble silver) have a higher tendency to produce argyria than metallic silver [29].

After reviewing 70 cases of argyria associated with ingestion of silver and silver compounds [30], the EPA established a chronic oral RfD of 5 μg/(kg day), or about 350 μg for a 70 kg person [31]. The amount of silver contained in the dose of DSF applied intraorally in this study exceeded the RfD for all subjects, with 1 subject exposed to nearly 9 times the RfD. However, the RfD represents a conservative estimate of silver that can be ingested every day of one’s entire life and presumably not result in the development of argyria or argyrosis. Allowable short-term exposure (1–10 days) of 1.142 mg of silver per liter of drinking water was proposed by the EPA in May 1989 [26]. Based on argyria development reported by Gaul and Staud [30], the EPA set the lifetime lowest allowable effect level (LOAEL) for silver exposure at 1 g total dose, which is well over 400 times the maximum amount of silver applied in this study.

Most case reports of argyria indicate a pattern of chronic ingestion of high concentrations of colloidal or soluble silver, with total exposure in the 1– 30 g range [32]. Evidence from a controlled study of the use of chewing gum containing silver acetate as a smoking deterrent would suggest significantly higher concentrations of silver exposure can be tolerated daily over several months without development of argyria or argyrosis. On average, 21 participants in the study by Jensen and colleagues [33] chewed 31.9 pieces of gum per week (4.5 pieces/day) for up to 12 weeks. Each piece of gum contained 6 mg of silver acetate (approximately 3.9 mg silver ion) and chewing for 30 minutes was reported to release about half of the silver from the gum (approximately 1.9 mg silver ion). Chewing the average number of pieces each day would have resulted in ingesting 8.6 mg silver ion per day for each day of the 12-week study. Some subjects reported chewing as many as 63 pieces of gum per week (9 sticks/day average) during the first 2 weeks of the study, giving silver exposure as high as 17.1 mg per day. Physical examinations found no signs of discoloration in the oral mucosa, teeth, skin, or eyes at any time up to 6 months after the study. Of significance, skin biopsies were assessed to be normal by hematoxylin and eosin staining, and autometallographic silver development found only a few traces of silver in the majority of skin biopsies after participation in the study. Examination of skin biopsies pre- and post-silver gum treatment, found that only a ‘few’ biopsies showed increased silver grains after completion of the silver gum treatment, compared with ‘sparse’ silver grains seen pre-treatment.

The controlled, chronic silver exposures reported by Jensen and colleagues [33] significantly exceed the dose and number of applications associated with the therapeutic use of DSF. For example, a 70 kg person chewing the average (4.5 pieces) or highest (9 pieces) number of gum pieces per day would exceed the RfD for silver by nearly 25 to 50 times, respectively. The fact that argyria or argyrosis did not develop with these higher exposures suggests it is unlikely either condition will develop from one-time or occasional use of DSF with an exposure to silver that is 2.5 to 5 times less. Moreover, there was no staining of teeth or irritation of the oral soft tissues reported at these recurring exposure concentrations over 12 weeks.

The pharmacokinetics of serum silver in 4 of 6 subjects treated with DSF in this study suggested maximum serum concentrations of silver occurred within 1–3 h after treatment. Apparently, delayed ingestion of DSF in the other 2 participants resulted in a delayed maximum serum silver level until the end of the monitoring (4 h after DSF application) in 1 participant, and no obvious serum silver peak in the other. For future studies examining the serum pharmacokinetics of a topically-applied, aqueous compound such as DSF, we suggest utilization of a plain water ‘swish and swallow’ immediately after application to facilitate dependable clearance and ingestion of excess product from the oral cavity. With these caveats, the mean maximum serum silver level was 206 nmol/L, with no participant over 270 nmol/L. These serum silver concentrations are well below those reported to be without toxic effect in subjects chewing silver acetate-containing gum [33]. After two weeks of gum chewing, the mean serum silver in the Jensen study was 512 nmol/L, decreasing to 406 nmol/L at week 6, and 387 nmol/L at week 12. The serum silver level was essentially maintained above 371 nmol/L for a period of at least 10 weeks without development of argyria or argyrosis. These results suggest, again, that the lower serum silver level associated with a single application of DSF at the dose used in this study should not induce a toxic effect.

This was a preliminary study and, as such, the number of subjects was limited. In addition, the follow-up period was shorter than optimal, given likely delayed clearance and ingestion of DSF from the oral cavity. As a result we were not able to calculate the Area Under the Curve (AUC) for this exposure. Despite these limitations, the data obtained are certainly useful in preliminarily assessing safety and planning future studies.

## Conclusion

The short term results suggest serum concentrations of fluoride and silver after topical application of DSF should pose little or no toxicity risk when used in adults and provides guidance to investigators as they work to more fully characterize its safety profile. When applied by a professional with appropriate care, DSF is likely safe. The gingiva and oral mucosa are not inflamed, ulcerated or pigmented.

## Competing interests

The funders had no role in study design, data collection and analysis, the decision to publish, or preparation of the manuscript. P. Milgrom is a principal in ADP Silver Dental Arrest, LLC. The other authors declare no competing interests.

## Authors’ contributions

EV, GZ, and JLC helped organize the study, gain IRB approval and recruit and manage participants. EC collected the blood and managed the participants. JLC participated in the IRB process, applied the silver fluoride, and served as an examiner. DRT analyzed the fluoride. GEW participated in the data analysis and was the primary author of the discussion. RD analyzed the silver. LLM served as the project statistician. PM carried out some of the clinical activities and wrote the first draft of the manuscript. All the authors participated in and approved the final version of the manuscript.

## Pre-publication history

The pre-publication history for this paper can be accessed here:

http://www.biomedcentral.com/1472-6831/12/60/prepub
